# Hospital Reform

**Published:** 1897-11-20

**Authors:** 


### HOSPITAL REFORM.

### THE SPEECH THE "LANCET" CRITICISED.

What Sir Henry Burdett Actually Said.

The following is a verbatim transcript of the shorthand writer's notes taken at the meeting of the Hospital Reform Association, held at St. Martin's Town Hall on October 21st, 1897, of the remarks made by

Sir Henry Burdett: My Lord, Ladies, and Gentlemen,—It seems to me that all these proposals to form yet another committee, to make more inquiries, to go over the old ground, to take up the old facts, and to report on the same lines as the committees that have gone before, is not the way to solve the out-patient question at all. I believe that the time has entirely gone by for all that, and I am old enough and I have worked long enough among the hospitals to believe that we shall never get a better investigation and a more impartial one, one which is more enlightening to all intelligent men who have at heart the well-being of the medical profession and of those who attend our hospitals, than that report of Sir William Fergusson's Committee in 1870 which I have read in the last few days. Those who go over that report and go over the evidence which was embodied in the various documents which accompanied it—the reports of the smaller committees—and who have a knowledge of the working of hospitals to-day and the different and conflicting points which attach to the out-patient question, must agree that they have in these documents every material fact which any committee can bring out. I am strongly opposed by conviction, and as a practical man who has a keen sense of the value of time, to yet another committee of investigation into the out-patient department of our hospitals. I think that if you do have a committee at all it should not be one for investigation, but should stand upon the old facts, which none of us dispute, and should take a new departure by bringing together those who have it in their power to bring about a new system and new methods in the treatment of disease in our out-patient departments. That is the principle on which I stand, and I am bound to say, as a practical man, that statements like we have heard to-day, the truth of which far be it from me to question, that out-patients are seen hurriedly, that the out-patient departments are so crowded, and that poor young doctors are so different from other young Englishmen that they break down after four hours' work in that department, and are overburdened, should not be so much insisted upon.

As a matter of fact the only material difference for our purpose in this out-patient question to-day to what it was in the day of Sir William Fergusson's Committee in 1870, 27 years ago, is that the out-patients who now flock to the hospitals are at least three times more than they were then. Therefore, from the point of view of the system, we are bound as practical men to say that the people who use the out-patient department find it in some way, or for some purpose, better than other means which they are offered for the treatment of ailments and disease when they fall ill. I think that that is the position that the medical profession has to face. They have got to make the treatment and attendance that the general practitioner gives more attractive and more efficient. I ask, can any hospital physician or hospital surgeon deny that there are a great number of cases which come to them at a later period of their disease which are in a condition that they ought not to be in if they had had adequate medical treatment? This Hospital Reform Association is, as I understand it, an association of medical men, and although I have not the honour of being a licensed member of the medical profession, still I have had a good deal to do with medicine, and have gone through the curriculum of medicine, and I do say to you, the medical profession, that the first thing to do is to recognise the plain facts that after you have put down on one side of the paper every evil which attaches to the out-patient department and every reason which would make a wise man or woman not wish to go there when they were ill, still the fact remains that the people do go there, and that the numbers continue to grow out of all proportion to the annual increase in the growth of the population. That being the case, I think, as a practical man, that there cannot be any doubt that the cause of this is due to a want of full utilisation of the opportunities which hospitals offer, not only to the laity but to the medical profession, and that we must go really into this question of the treatment of the patients outside as well as inside the hospitals. If you want to get this question really faced you must look at it absolutely from that point of view, and I should like myself to see that the post graduates, the men who are labouring among the poor as medical men in a large and crowded town like London, should have full facilities for going to the hospitals and attending post graduate classes without any payment at all. I think, if the consulting staffs were to give these facilities, that, as more medical men utilised them, so you would get fewer and smaller demands on the out-patient departments.

But the whole question of the out-patient department, as far as it affects hospitals, is one which we have to consider to-day, and I am strongly of opinion that there is one point on which a committee, if it investigated the subject now, would arrive at a different conclusion to that of Sir William Fergusson's Committee. I allude to the question of allowing patients to pay something for their treatment when they go to the hospital. You know, of course, that Sir William Fergusson's Committee admitted that it was sound in principle to allow a man to have medical relief for a small sum if he joined a provident dispensary. Now Mr. Mocatta and Mr. Holmes have worked very hard for many years, and with some success, on this question of provident dispensaries in London, but somehow or another it has never really "caught on" to anything like the extent we might have hoped it might have done, having regard to the people who want medical aid. I have, therefore, come to the conclusion that we ought to arrive at some system which would enable the hospitals to say, "We will only give relief to those people who are unable to pay for medical attendance, unless the nature of their case is such that they require the medical attendance which they can only get at a hospital, and that if they want such assistance (their position being above the level of those who ought to have such relief) they must show that they have continuously and regularly given according to their means to the hospitals." Now, of course, that is only another way of applying the provident principle. It is working the provident principle through the out-patient department. I have attended the out-patient departments lately to see whether there was much difference in the social position of the patients who go there. I did not think that there was much difference, but there is this great fact, that, taking the people who attend as a whole, there is no doubt they can all of them, with the exception of perhaps 10 per cent., pay something, if they choose, for the medical treatment which they receive. I also think, from information which I have gathered from the interviews I have had with these people, and from hearing the answers to and the way in which they treat the registration officers of the hospitals, that they would welcome a system by which the so-called provident dispensary idea could be adopted in the hospital, so as to enable them to show that they were willing, quite apart from the fact that they were ill, to contribute regularly as subscribers to the hospitals of London. Following out that view I hope that before long an important departure will be taken in connection with some of the central bodies who will by a decision, which I hope they will come to, induce every hospital to have a registration officer in the outpatient department who shall inquire from each out-patient as to his social position before he is permanently admitted to relief. If that is done there should be very little difficulty, by the possession of a subscription book for small subscriptions, on the system which is known as the Prince of Wales's Hospital Stamps, in preventing many of the evils which attach, and have attached in the past, to the collection of small subscriptions. We might thus make every man and woman able to do so subscribe quite regularly, and always have in a handy form, which they could produce at any time, evidence of the fact that they are subscribers to the hospitals.

I really hope that very grave consideration will be given to the question before this conference consents to appoint another committee to go over the old ground. I think it would better if we were to adjourn for a little while, say. to the beginning of next year, so that they might understand exactly what is the outcome of this, the Diamond Jubilee year, in which they have had new departures in reference to the collection of money and in which we may possibly have new departures as to the distribution of money among the hospitals. You cannot force this question and it is not to be settled in a day, nor a year, nor possibly in ten years. I do, however, believe that, having regard to the continual proddings of Dr. Glover and to the influence of other people all round, backed by the knowledge which I have, if you could see your way, at any rate not to come to a decision to appoint a committee to-day, but to delay a decision as to that until you see the result of this year with reference to the out-patient department, you will later on think with me that that decision was a right one, and that it offered you a solution to this question.

Mr. George Brown: Do I rightly understand you to suggest that the hospital patients should bring with them Jubilee stamps in order to receive medical treatment, and that this would give them the right to free medical relief?

Sir Henry Burdett: No, I made no such suggestion. I should object to that very strongly. I only wished that out-patients should be able to produce these stamps as evidence of their feeling towards the hospitals, and of the fact that they were willing to do their best to subscribe to them.

Mr. George Brown: Where are these stamps to be produced? Are they to be exhibited at the street corners?

Sir Henry Burdett: If a man under the present system is a subscriber to a hospital he gets so many tickets. I am in favour of free medical relief, but if it is right for a poor man to give 4s. 4d. a year to a provident dispensary and to receive medical treatment at that dispensary there can be no harm in that man giving voluntarily half-a-crown or so to a hospital, and if he wants relief, and he is a proper case for free treatment, going to a hospital and saying that he has done his best for the hospitals. The possession of these stamps would be evidence of this fact.

Sir Henry Burdett, later on, in consequence of a misunderstanding of the foregoing speech by subsequent speakers, rose and said: May I say a word in explanation? I wish this meeting to be under no misapprehension as to the meaning of my reference to general practitioners. I do not think that there is anybody in this room more indebted to the general practitioner than I am. I quite believe in him, and recognise that he is the backbone of the medical profession, and that on his efficiency the welfare of our homes is dependent. All I wanted to bring out is this, that as a matter of fact, in the enormous body of men in general practice, especially among the class of men these out-patients go to when they do not attend the hospitals, there are a large number who would be infinitely helped, both as to their practice and reputation, if they could go to the hospitals and attend post-graduate classes.

